# Ultrasound wrist mapping to develop a noninvasive radiation detector for dynamic positron emission tomography

**DOI:** 10.1038/s41598-026-39073-7

**Published:** 2026-02-08

**Authors:** Marc-Antoine Leclerc, Mihai Mesko, Youstina Daoud, Shirin A. Enger

**Affiliations:** Medical Physics Unit, Department of Oncology, Faculty of Medicine, McGill University, 1001 boul Décarie, Montreal, H4A 3J1 Quebec Canada

**Keywords:** Dynamic PET, Arterial input function, Radiation detector, Ultrasound, Anatomy, Health care, Medical research

## Abstract

Quantitative PET studies require a measurement of the arterial input function (AIF), the time-dependent radiotracer concentration in arterial blood plasma. Several groups are developing non-invasive detectors to measure the AIF from the radial artery. This study quantifies the depth and cross-sectional area of the radial artery and accompanying veins at different wrist positions using ultrasound. These anatomical data will guide the design of a non-invasive, wrist-worn detector for AIF acquisition—a practical, patient-friendly alternative to invasive blood sampling. Ultrasound imaging of the wrist was performed on 154 healthy individuals at specified distances from the distal wrist crease (2 *cm*, 4 *cm*, and 6 *cm*). The depths of the radial artery at distances of 2 *cm*, 4 *cm*, and 6 *cm* from the distal wrist crease are 3.36 (1.25) *mm*, 4.08 (1.81) *mm*, and 4.66 (2.23) *mm*, respectively (mean (SD)). Similarly, the cross-sectional areas of the radial artery at these distances are 4.23 (1.75) *mm*$^{2}$, 3.92 (1.71) *mm*$^{2}$, and 3.90 (1.88) *mm*$^{2}$, respectively. The radial artery becomes larger and more superficial near the wrist, suggesting a radiation detector be placed 2 *cm* from the distal wrist crease on the left arm, where it is generally more superficial than on the right.

## Introduction

Positron emission tomography (PET) is a nuclear imaging modality that allows the study of biological processes of a radioactively labelled molecule (radiotracer). In a dynamic PET scan (dPET), quantitative measurements of the radiotracer’s biochemical properties, such as its uptake and clearance rate in tissues of interest, are possible^1^. Some uses of these measurements include the evaluation of tumor treatment response, the detection of cancer recurrence and the development of new radiotracers^2–4^.

Analyzing the dPET data to extract the quantities of interest necessitates quantitative models of the radiotracer’s kinematics. These models require a time measurement of the tracer’s activity concentration in the blood plasma called the arterial input function (AIF)^1^. The gold standard method for this measurement requires direct blood sampling. However, this is an invasive and complex procedure with several downsides, such as discomfort for the patient, the need for additional trained personnel and equipment, and radiation exposure from blood handling. Non-invasive methods for obtaining the AIF are available, but they have limitations that restrict their routine clinical adoption. For instance, population-based input functions represent averaged AIF measurements across large patient cohorts for a given radiotracer. Although they have shown good agreement with blood sampling, they cannot be used when researching novel radiotracers since no prior data set exists. They can also introduce a small bias for patients with diseases that affect the kinetics of the tracer^5^. Image-derived input functions use the PET scanner to measure the AIF over a large blood vessel. The primary drawbacks of this technique are partial volume effects, which arise from the system’s limited spatial resolution. This is particularly challenging for brain studies, where the small size of the blood vessels makes the partial volume effects important^6^. External radiation detectors are being developed to measure the AIF non-invasively^7–16^. The most promising designs are miniature PET systems such as the one developed by Wei et al. (2022)^10^. However, this approach is unlikely to be cost-effective, considering the expensive crystal scintillator channels involved. Currently, the routine use of dPET for clinical applications has not been widely implemented, partly due to the lack of an accurate, non-invasive, and cost-effective method for measuring the AIF.

A non-invasive and cost-effective radiation detector remains a promising avenue for acquiring the AIF during dPET scans. Caroll and Enger (2020) developed a plastic scintillating fiber-based detector to obtain the AIF by measuring activity from the radial artery^8,11^. The authors simulated the performance of their detector using a radial artery depth of $(1.99 \pm 0.99)$ *mm* based on the anatomical measurement in the study by Lee et al. (2016)^17^. Following this, Daoud et al. (2023) developed a graphical user interface that imports ultrasound wrist measurements for use in the Monte Carlo simulation of the detector described by Caroll and Enger (2020)^18^. This simulation estimates the expected response of the detector based on the specific patient’s wrist anatomy. In particular, the anatomical data are used to derive coefficients that relate the detector signal to radioactivity. For instance, assuming that the detector counts originate solely from the radial artery, the AIF can be expressed as:

1 $$\begin{aligned} AIF = C \cdot \epsilon \end{aligned}$$

where *C* ($\frac{{\textrm{counts}}}{{\textrm{s}}}$) is the count rate of the detector corrected for efficiency and dead-time, and $\epsilon$ ($\frac{\text {kBq} \cdot \text {s}}{\text {mL} \cdot \text {count}}$) is the coefficient relating the count rate to the AIF, obtained from simulations. Equation 1 can be extended to account for multiple sources of radioactivity to more accurately reflect the detector’s measurements in practice; this will be presented in future work.

The accuracy of the coefficient $\epsilon$ depends on how well the simulation models the experimental geometry, which in turn relies on precise measurements of the patient’s wrist anatomy. For example, the performance of the detector is hypothesized to depend on the depth of the radial artery, as deeper vessels result in greater radiation attenuation. Similarly, the cross-sectional area of the artery influences performance, since larger vessels can carry more radioactivity, improving counting statistics. Therefore, to evaluate detector performance across a population, simulations incorporating diverse wrist anatomies are essential.

Moreover, several research groups are developing non-invasive AIF detectors targeting wrist vasculature^12–16^, and would benefit from a more detailed anatomical characterization of the wrist, specifically measurements of the depth and cross-sectional area of the radial artery and veins at various positions along the forearm. Again, understanding inter-patient variability in wrist anatomy is important for evaluating and optimizing detector performance in simulation studies. In addition to detector development, such anatomical information is valuable for various medical procedures, including radial artery cannulation^17,19–23^.

This study aimed to measure the depth and cross-sectional area of the radial artery and the radial venae comitantes in healthy individuals. The collected data will be used in a future simulation study aimed at characterizing the detector’s performance across different wrist geometries. Additionally, this future work will investigate other factors that may influence performance, such as the impact of detector misplacement relative to the radial artery and the potential contribution of tracer flow in surrounding veins. In practice, an ultrasound imaging session will be conducted prior to each dPET scan to calculate a patient-specific calibration factor for the detector.

## Methods

A total of 154 healthy individuals were recruited to participate in the study. Exclusion criteria included the presence of chronic illnesses or any symptoms or discomfort at the time of recruitment. The study objectives were clearly explained to all participants, and written informed consent was obtained prior to participation. Ethical approval was granted by the Medical/Biomedical Research Ethics Committee of the *Centre intégré universitaire de santé et de services sociaux* of West-Central Montreal Health (study number 2022-2733). The research methods were performed in accordance with the Declaration of Helsinki. Research participants provided informed consent for publication of the images presented in this work and sex differences were accounted for in the study design. Participant demographic and physical characteristics, including handedness, sex, age, weight, and height, were recorded. To ensure full demographic data availability (age, height, and weight), the final sample for the primary anatomical analysis was established at 147 participants. While this entire cohort was used to assess general demographic effects to maximize statistical power, handedness data was documented for a subset of 96 participants. Consequently, analyses specifically assessing the impact of handedness were restricted to participants with recorded handedness data. A summary of this information is presented in Table 1.

**Fig. 1:**
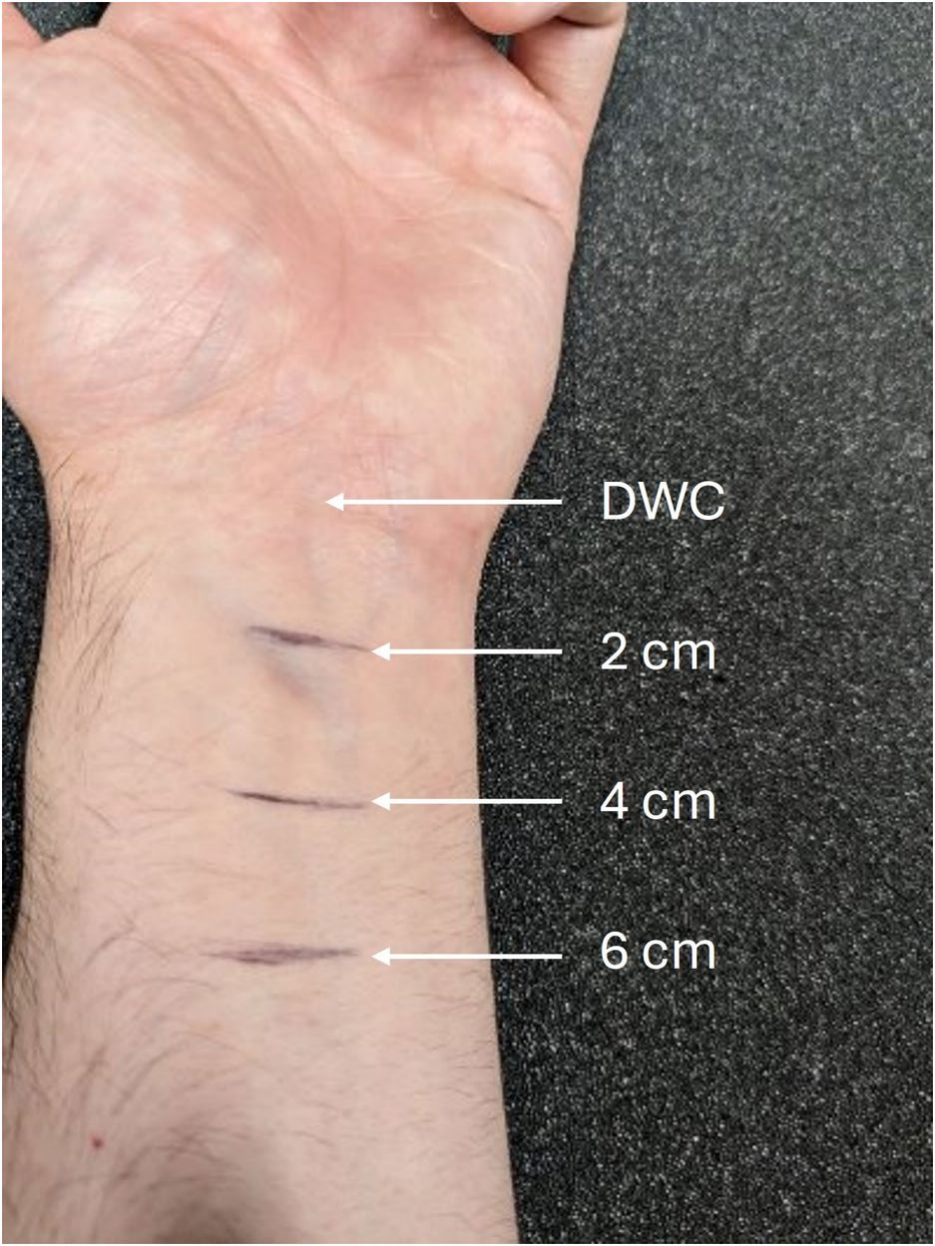
Participant’s left arm. The supinated arm rested on a flat surface. Three lines were drawn at 2 *cm*, 4 *cm* and 6 *cm* from the DWC. Cross-sectional ultrasound imaging was performed at these three lines.

The patient’s left arm was placed on a flat surface in a supinated position. The distal wrist crease (DWC) was identified, and three lines were drawn at 2 *cm*, 4 *cm* and 6 *cm* from the DWC as shown in Figure 1. An ultrasound gel (Wavelength High Viscosity Gel, National Therapy Products Inc.) was applied to the skin to cover all three lines. A 12 *MHz* wide linear array transducer (13L4w, BK Medical) connected to an ultrasound system (bk3000, BK Medical) was used to image the forearm.

**Fig. 2:**
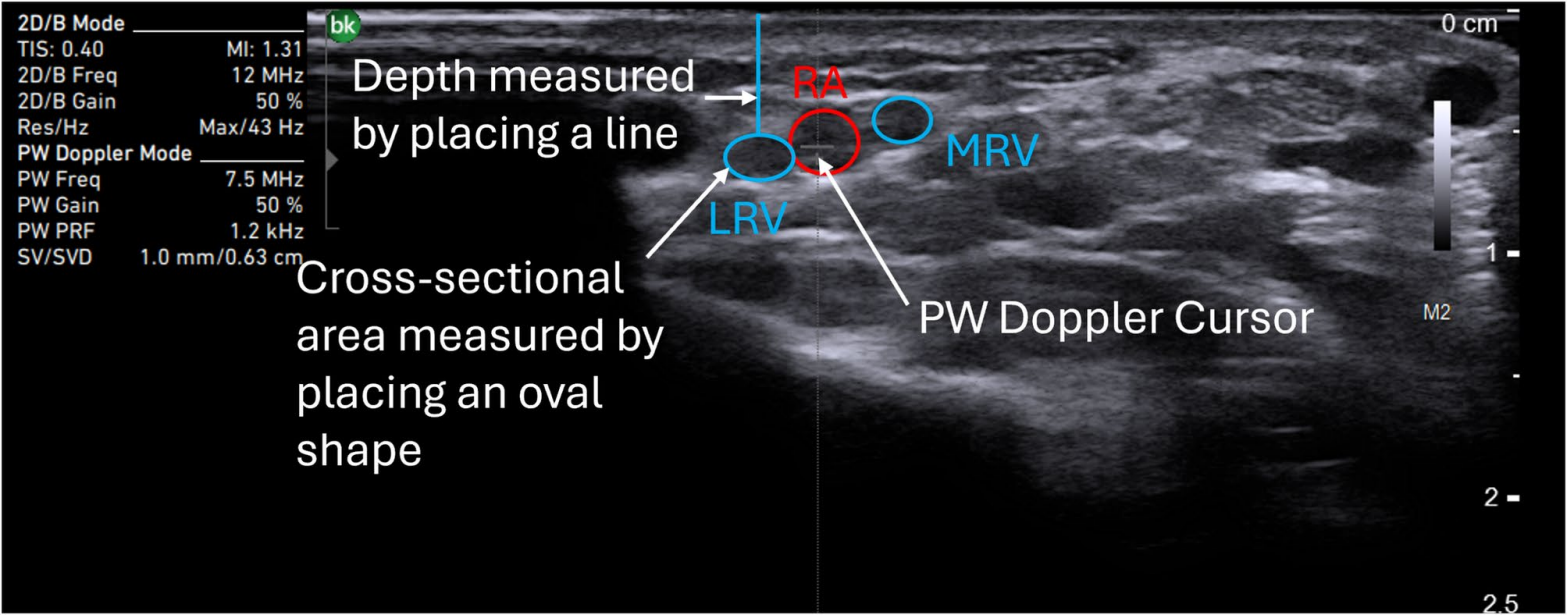
Cross-sectional view of the left forearm at the 2 *cm* line. LRV is the lateral radial vein, RA is the radial artery, and MRV is the medial radial vein. The radial artery was identified by a cardiac waveform from the PW Doppler mode. The lateral and medial radial veins are the black circular shapes next to the radial artery. The depth of the radial artery and the lateral and medial radial veins was measured by drawing a line from the skin surface to the outer wall of each blood vessel on the image. This line was used to quantify the spatial distance between the skin and the vessel. Similarly, the cross-sectional area of each vessel was measured by placing an oval shape over the visible cross-section of the vessel in the image, matching its contours as closely as possible. An example is shown for the lateral radial vein.

At the 2 *cm* line, the probe was applied with the minimum pressure needed to obtain a 2-D image in B-mode. The radial artery was found using a cross-sectional view and identified using pulsed wave (PW) Doppler mode with the cursor centered on the radial artery as illustrated in Figure 2. Regular peaks corresponding to cardiac cycles in the PW Doppler waveform were considered evidence for the radial artery. The radial venae comitantes, herein called the lateral radial vein and the medial radial vein, were identified as circular shapes next to the radial artery. The image was frozen and exported for later analysis to minimize the time the participants needed to spend in the study. This image acquisition was repeated at the 4 *cm* and 6 *cm* lines. Measurements were repeated on the right arm.

Image segmentation was performed using the ImageJ software version 1.54^24^. Pixel size calibration was conducted using the scale bar provided on the ultrasound image, as illustrated in Figure 2. Cross-sectional ultrasound images were analyzed to measure the depth and cross-sectional area of the radial artery, as well as the lateral and medial radial veins. Depth was measured as the linear distance from the outer skin surface to the inner edge of the vessel wall. This was done in ImageJ by manually placing a line between these two anatomical landmarks. To assess the cross-sectional area, an oval was manually adjusted to match the shape of the vessel lumen, and the enclosed area was calculated using ImageJ’s built-in measurement tools. An example of this process is shown in Figure 2 for the lateral radial vein.

Data analysis was performed using Python (version 3.13.5) with the pandas (version 2.2.3) and statsmodels (0.14.4) libraries. To determine the predictors of blood vessel depth and cross-sectional area, linear mixed-effects models were employed. This modeling approach was selected to account for the within-subject correlation arising from repeated measurements (both left and right arms measured for each participant) and to robustly handle missing data points without discarding entire participants.

To optimize statistical power relative to data availability, the analysis was conducted in two stages. First, a primary model was fitted to the maximal available cohort ($N=147$) to assess anatomical and demographic predictors. The model structure was:

2 $$\begin{aligned} \begin{aligned} Y_{i,j}&= \beta _0 + \beta _{\text {side}}(\text {Arm Side}) + \beta _{\text {dis}} (\text {Distance from DWC}) + \beta _{\text {sex}}(\text {Sex}) + \beta _{\text {age}}(\text {Age}) + \beta _{\text {BMI}}(\text {BMI}) + \mu _i + \epsilon _{i,j} \end{aligned} \end{aligned}$$

Second, a secondary model was fitted to the sub-cohort with documented handedness records ($N=96$) to explicitly test for handedness and arm dominance effects. This model extended the primary structure as follows:

3 $$\begin{aligned} \begin{aligned} Y_{i,j}&= \beta _0 + \beta _{\text {side}}(\text {Arm Side}) + \beta _{\text {hand}}(\text {Handedness}) \\&\quad + \beta _{\text {dom}} (\text {Side} \times \text {Handedness}) + \beta _{\text {dis}} (\text {Distance from DWC}) \\&\quad + \beta _{\text {sex}}(\text {Sex}) + \beta _{\text {age}}(\text {Age}) + \beta _{\text {BMI}}(\text {BMI}) + \mu _i + \epsilon _{i,j} \end{aligned} \end{aligned}$$

where $Y_{i,j}$ is the *j*-th outcome variable (blood vessel depth or cross-sectional area) for the *i*-th participant. Both models included a random intercept $\mu _i$ for each participant and a residual error term $\epsilon _{i,j}$.

Linear coefficients $(\beta _0, \beta _{\text {side}}, \beta _{\text {hand}}, \beta _{\text {dom}}, \beta _{\text {dis}}, \beta _{\text {sex}}, \beta _{\text {age}}, \text {and } \beta _{\text {BMI}})$ are reported as the estimated change in the outcome variable relative to the reference group: right arm (Arm Side), right-handed (Handedness), 2 cm (Distance from DWC), and female (Sex). For quantitative variables (Age, BMI), coefficients represent the change per unit increase (years, $kg/m^2$). In the secondary analysis, an interaction term ($\beta _{\text {dom}}$) was included between arm side and handedness to rigorously test for an arm dominance effect. The significance of all fixed effects was assessed using Wald Z-tests at a 5% significance level ($P \le 0.05$).

## Results

The results of the depth and cross-sectional area measurements obtained from the cross-sectional images are summarized in Table 2. Data are presented as mean (SD). On average, the depth of the radial artery, as well as the lateral and medial radial veins increases with distance from the DWC. In contrast, cross-sectional area measurements tends to decrease as the distance from the DWC increases.

**Fig. 3:**
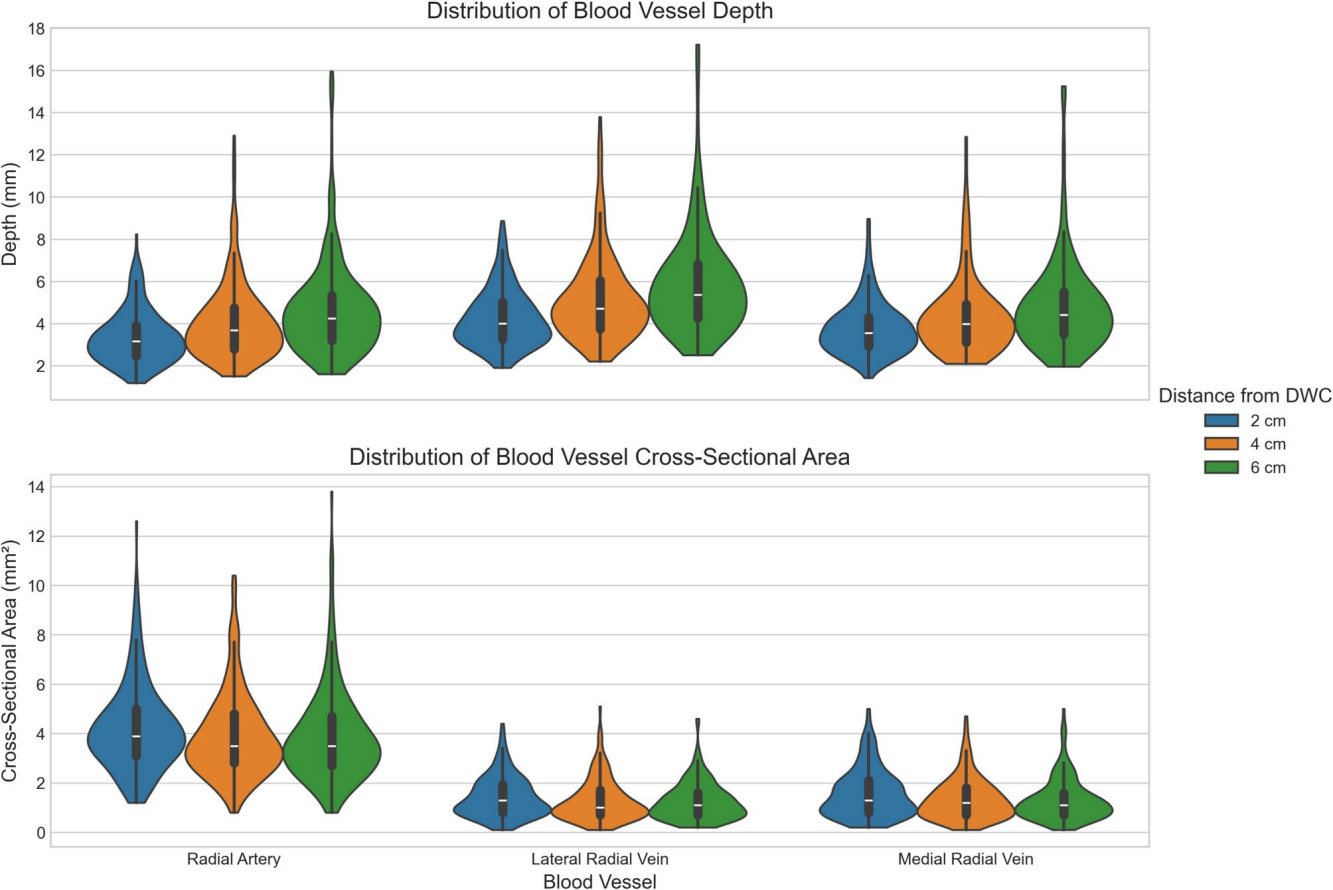
Distribution of blood vessel depth and cross-sectional area as a function of distance from the DWC, complementing Table 2. Violin plots with embedded box-and-whisker elements illustrate an increasing trend in vessel depth and a decreasing trend in cross-sectional area with distance. One observable effect in the plots is that patients with notably deep and large blood vessel skew the distribution toward higher values.

Figure 3 illustrates the distribution of blood vessel depth and cross-sectional area as a function of distance from the DWC, complementing the data presented in Table 2. Violin plots are used to visualize the data distribution, with embedded box-and-whisker plots providing additional insight by showing the median and quartiles. The plots illustrate the increase of vessel depth with distance and the corresponding decrease of vessel cross-sectional area. Moreover, participants with notably high values for the depth and area appears to skew the distribution upward.

The depths and cross-sectional areas of the vessels were also analysed with respect to demographic and anatomical parameters, as presented in equation 2. Table 3 shows the results of the primary linear mixed model for blood vessel depth. The $\beta$ coefficient represents the estimated change relative to the reference group (right arm for Arm Side, 2 *cm* for Distance from DWC, fermale for Sex). For example, a negative $\beta$ for Sex (Male) indicates that males had lower values for vessel depths than females. A significant difference in depth was found between the right and left arms for all blood vessels ($P < 0.001$), with the right arm demonstrating greater depth. Measurement position was a strong predictor of depth for all blood vessels ($P < 0.001$), with depth increasing as the distance from the DWC increased; this supports the descriptive data in Table 2. A statistically significant difference in vessel depth was also observed between males and females, with females exhibiting greater average depth ($P < 0.001$). Across all blood vessels, depth was strongly and positively associated with BMI ($P < 0.001$). Age was not found to be a significant predictor of vessel depth.

Table 4 presents the results of the primary linear mixed model for blood vessel cross-sectional area. A significant difference between arm sides was observed only for the lateral radial vein ($P = 0.012$), which displayed a larger cross-sectional area in the left arm. Distance from the DWC was also a strong predictor of cross-sectional area for all vessels ($P \le 0.011$), consistent with the data in Table 2. Moreover, males exhibited significantly larger vessel cross-sectional areas compared to females ($P < 0.001$). Age and BMI were positively associated with the cross-sectional area of the radial artery ($P = 0.004$ and $P = 0.048$ respectively).

Table 5 presents the results of the secondary linear mixed model (equation 3) assessing the impact of handedness and arm dominance (the interaction term) on blood vessel depth using the sub-cohort ($N=96$). No statistically significant effects were observed for either handedness or the interaction term across any of the vessels studied. The associations with arm side, measurement position, sex, age, and BMI remained consistent with the findings from the primary analysis (Table 3).

Similarly, Table 6 details the results for cross-sectional area, where neither handedness nor arm dominance acted as significant predictors. A notable deviation from the primary analysis (Table 4) was the loss of statistical significance for BMI in this sub-cohort. This discrepancy is likely attributable to reduced statistical power in the secondary analysis ($N=96$) compared to the primary analysis ($N=147$); consequently, the primary model (Table 4) should be considered the more robust estimator for demographic covariates such as BMI.

**Fig. 4:**
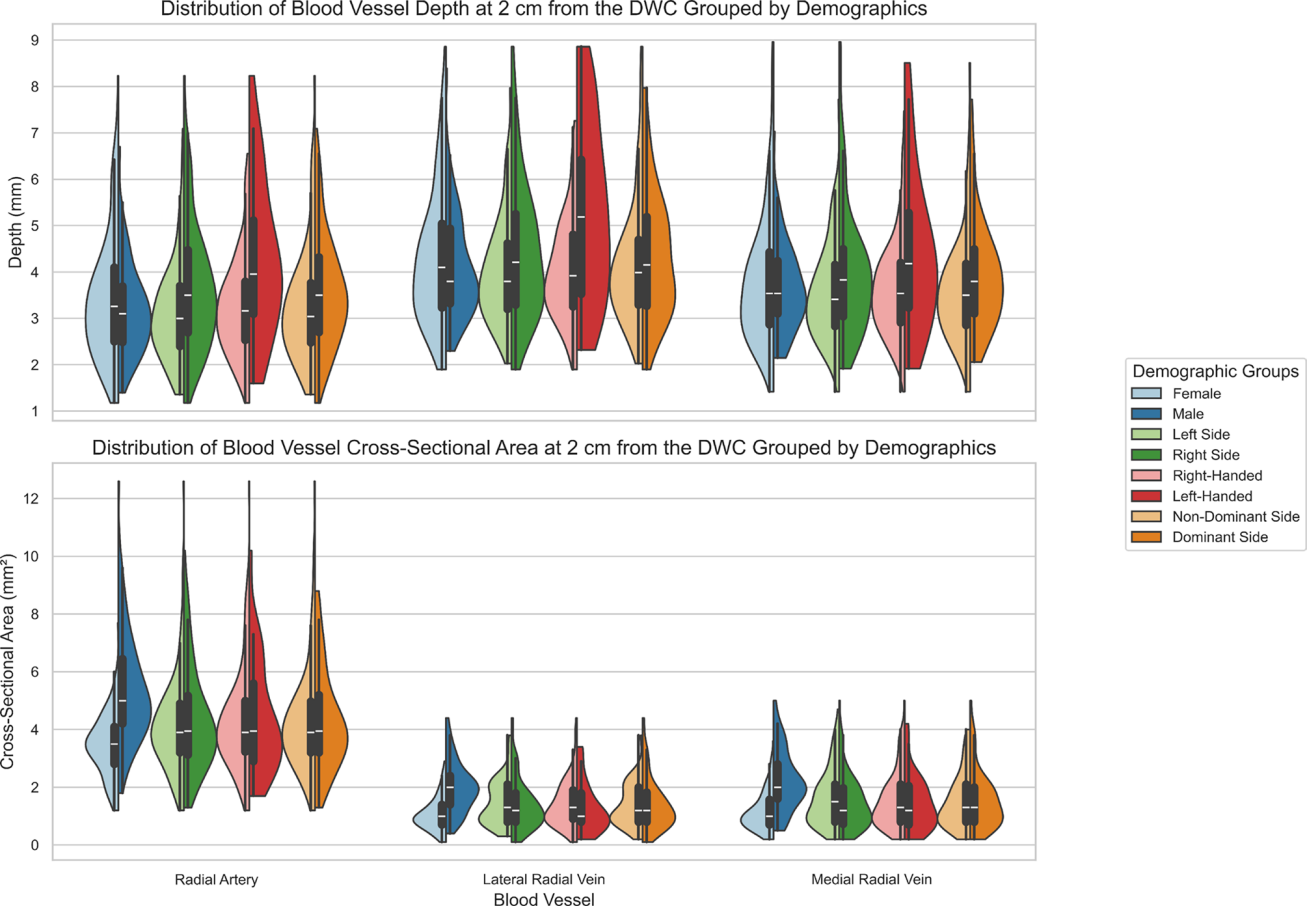
Violin plots illustrating the distribution of blood vessel depth and cross-sectional area across sex, arm side, handedness, and arm dominance. These visualizations expand upon the trends summarized in Tables 3, 4, 5 and 6, highlighting the asymmetries. Box-and-whisker overlays reveal upward skewness in the data, similar to what was observed in Figure 3.

Figure 4 complements Tables 3, 4, 5 and 6 by showing the distribution of blood vessel depth and cross-sectional area, grouped by sex, arm side, handedness and arm dominance. The distributions are shown with violin plots and the central tendency and skewness can be visualized with the box-and-whisker plots. Similar to Figure 3, the distributions are skewed upward, suggesting potential outliers with very deep and large blood vessels.

## Discussion

For many years, research groups have explored the development of wrist-worn radiation detectors to non-invasively measure the AIF in quantitative PET studies^7–9,11–16,18^. The wrist, and specifically the radial artery, presents an attractive target due to its superficial location, accessibility, and relatively consistent anatomical position across individuals. These characteristics make it well-suited for wearable detector placement, reducing the complexity of alignment and calibration. A detailed understanding of the vascular anatomy in this region is important for optimizing detector design, particularly with respect to signal accuracy, attenuation correction, and robustness to anatomical variability. This study aimed to quantify the depth and cross-sectional area of the radial artery, as well as the lateral and medial radial veins, at various wrist positions using ultrasound. These parameters, along with their inter-patient variability, will be incorporated into Monte Carlo simulations to aid in the design, development and calibration of a practical, non-invasive AIF detector. Such a device could serve as a patient-friendly alternative to invasive blood sampling, improving the feasibility and comfort of quantitative PET imaging in both research and clinical settings.

Results indicate that the radial artery becomes more superficial as it approaches the wrist, with the shallowest depth observed at 2 *cm* from the DWC, as shown in Table 2. This suggests that positioning the sensitive volume of an AIF detector at this location may minimize photon attenuation and scatter, thereby enhancing detection accuracy. Additionally, the cross-sectional area of the radial artery increases closer to the wrist, which has two implications. First, a larger artery cross-section reduces the spatial resolution requirements of the detector, second, it increases the volume of radiolabeled blood passing through the region, increasing emitted radiation and potentially improving the signal strength. Collectively, these anatomical characteristics support placing the detector approximately 2 *cm* from the DWC to optimize performance.

These findings could also suggest that the radial artery may be more superficial at the DWC and thus, placing the detector directly at the DWC could even be better for the detector performance. However, near the DWC and in the hand, the vascular anatomy becomes more complex. The radial and ulnar arteries bifurcate to form other arteries such as the superficial and deep palmar arches. While the radial and ulnar arteries generally follow a straight path along the forearm, the palmar arches exhibit curvature within the hand, complicating anatomical modelling. Additionally, the presence of carpal bones at the wrist limits deep ultrasound penetration, thereby reducing the visibility of underlying structures. Although the radial artery may be more superficial near the DWC, this increase in vascular complexity and the addition of bony structures introduce uncertainties that could be avoided. Therefore, it is hypothesized that positioning the detector slightly more proximally along the forearm, at 2 *cm* from the DWC may provide more favourable and predictable experimental conditions.

Further analysis presented in Table 3 showed that the radial artery tends to lie deeper in the right arm (depth at 2 *cm* from the DWC: 3.66 ± 1.39 mm) than in the left (3.17 ± 1.12 mm), implying that detector placement on the left arm may be more favorable. Other characteristics examined—such as sex, age, and BMI—were found to influence vessel anatomy but are fixed traits and therefore not actionable for optimizing detector placement. Also, although Figure 4 visually suggests that left-handed individuals and dominant arms possess deeper vessels, the secondary linear mixed model (Table 5) did not detect a statistically significant effect for handedness or the side $\times$ handedness (arm dominance) interaction. It is important to note that the small sample size of the left-handed group ($N=16$) inflates the standard error for these specific estimates. This reduction in statistical power increases the risk of a Type II error (false negative), where a true dominance effect may exist but goes undetected. Consequently, we interpret these null findings with caution.

The detector developed by Caroll and Enger aims to obtain the AIF by measuring the radioactivity concentration of the radiotracers flowing in the radial artery^8,11^. This is achieved by measuring photons from the electron-positron annihilation following a radioactive decay event, with the positron originating from a radiotracer in the radial artery. However, depending on how far the positron travels before annihilation and the depth of the radial artery, the detector may also measure positrons escaping the wrist, which will influence the signal.

Table 7, adapted from Champion and Le Loirec (2007)^25^, shows the maximum positron ranges in water for commonly used PET radioisotopes. In this study, the mean radial artery depth at 2 *cm* from the DWC was 3.36 *mm* (SD: 1.25 *mm*), suggesting that for the majority of individuals, positrons from ^18^F (with a maximum range of 2.63 *mm* in water) are unlikely to reach the detector directly. In contrast, positrons from other isotopes with longer ranges—especially ^82^Rb, which has a maximum range of 18.60 *mm* may reach the detector, potentially contributing to the signal alongside annihilation photons. This highlights the need to consider isotope-specific positron ranges during detector design and calibration. Another detection event that will contaminate the signal is scattered radiation. Scattered photons lose spatial information related to their origin because they violate the straight path assumptions. They will be accounted for by measuring the energy of the detected radiation and applying an energy window around 511 *keV* such that only non-scattered annihilation photons will be recorded.

The study by Domagała et al. (2021)^19^ is the most comparable to the present work. They measured radial artery depth and diameter at the styloid process and at 4 *cm* and 8 *cm* proximally. Their reported average depth at the styloid process (3.88 (0.86) *mm*) aligns closely with the depth measured in this study at 2 *cm* from the DWC (3.36 (1.25) *mm*). However, their radial artery diameter at the styloid process, when converted to a circular cross-sectional area (1.60 (0.39) *mm*$^{2}$), is notably smaller than the corresponding cross-sectional area found in this study (4.23 (1.75) *mm*$^{2}$). Additionally, Domagała *et al.* reported a decreasing artery diameter as it approached the wrist, which contrasts with the present findings in Tables 2 and 3, where radial artery cross-sectional area increased near the DWC.

These discrepancies may be explained by methodological differences, such as transducer-induced compression during ultrasound acquisition, which can distort vessel shape and affect the apparent cross-sectional area. Assuming a perfectly circular lumen may also oversimplify the artery’s morphology, particularly under varying pressure conditions. Moreover, Domagała et al. reported a mean participant age of 20 years, which is substantially younger than the mean age of participants in the present study (35 years). They also reported a mean BMI of 22.44, which is lower than the value presented in Table 1 (BMI of 25.0). As shown in Table 4, lower age and BMI are associated with a reduced cross-sectional area for the radial artery, which may partially account for the discrepancies observed between their findings and those of the present study. However, it remains unclear and difficult to explain why their study identified a decreasing radial artery diameter toward the wrist, whereas our findings indicate an increasing cross-sectional area of the radial artery in the same region.

Despite these differences, several findings from Domagała et al. are consistent with the present results. They observed that the right radial artery lies deeper than the left as well as significant size differences between sexes—findings that agree with Tables 3 and 4 in this study.

Table 8 summarizes radial artery depth and cross-sectional area data from other ultrasound-based wrist studies. Where necessary, reported diameters were converted to cross-sectional areas assuming a circular cross-section. The present results fall within one standard deviation of the means reported in most of these studies^17,19,23,26^. Two exceptions, the depth reported by Lee et al. (2016) and cross-sectional area reported by Domagała et al. (2021), differ by approximately two standard deviations and appear to be outliers relative to the broader literature. Overall, the anatomical measurements presented here are consistent with previously published data.

The studies summarized in Table 8 examined the depth and size of the radial artery. However, most did not specify the exact measurement position along the wrist^17,21–23,26,27^. Furthermore, none of the existing studies reported measurements for the depth or size of the radial veins. The present study addresses these gaps by providing detailed measurements of both the radial artery and the accompanying radial veins, with clearly defined anatomical reference points along the wrist.

This study has several limitations that should be acknowledged. First, there was an underrepresentation of participants aged 30 to 50 years, with only 32 individuals (21% of the cohort) falling within this age range. A more balanced age distribution would increase the generalizability of the findings to the broader population. Second, although no significant effects of handedness or arm dominance were observed, the statistical power of this secondary analysis was constrained by the availability records (reducing the sample size to $N=96$) and the low prevalence of left-handedness in the cohort ($N=16$). A study with a larger sample size for left-handedness data would be required to definitively rule out such effects. Third, participants’ weight and height were self-reported rather than directly measured, introducing potential bias due to inaccurate reporting. Future studies should collect these anthropometric data through standardized clinical measurements to improve accuracy. Lastly, the phase of the cardiac cycle was not controlled during ultrasound measurements. Since vessel depth and diameter can vary between systole and diastole due to changes in blood pressure, consistency in the timing of measurements relative to the cardiac cycle would reduce physiological variability and improve data reliability.

Future research will leverage the anatomical data collected in this study to design, simulate, develop, and calibrate an AIF detector based on the prototype developed by Carroll and Enger (2023)^8,11^. In parallel, a three-dimensional ultrasound imaging system will be developed to provide a more accurate and patient-specific representation of vascular geometry. This approach aims to move beyond the limitations of simplified cylindrical models and better reflect the complex anatomical variability of the radial artery and veins.

## Conclusion

This study found that the radial artery depth decreases while its cross-sectional area increases as it approaches the wrist. Therefore, to minimise attenuation and scatter, a radiation detector targeting the radial artery should be placed 2 *cm* from the distal wrist crease, where the artery depth is 3.36 (1.25) *mm* and the cross-sectional area is 4.23 (1.75) *mm*$^{2}$. Additionally, the detector should be positioned on the left arm, where the radial artery is generally more superficial.

**Table 1:** Demographic and physical characteristics of the study cohort included in the primary analysis (N = 147). Continuous variables are reported as mean (SD). Categorical variables are reported as count (percentage). Handedness data were available for a sub-cohort (N = 96) used in the secondary analysis.

Characteristic	Total	Males	Females
	(N = 147)	(n = 63)	(n = 84)
**Anthropometric Data**			
Age (years)	34.9 (14.4)	33.7 (13.8)	35.9 (14.7)
Height (cm)	169.9 (11.4)	178.8 (7.3)	163.3 (9.1)
Weight (kg)	72.5 (17.7)	82.6 (14.7)	64.8 (15.9)
BMI (kg/m$^2$)	25.0 (5.3)	25.8 (4.4)	24.4 (5.8)
**Handedness Data**			
Right-Handed	80 (83.3%)	31 (86.1%)	49 (81.7%)
Left-Handed	16 (16.7%)	5 (13.9%)	11 (18.3%)

**Table 2:** Overall results for blood vessel depth and cross-sectional area (N=147). Values are reported as Mean (SD).

Distance from DWC	2 cm	4 cm	6 cm
**Depth (mm)**			
Radial artery	3.36 (1.25)	4.08 (1.81)	4.66 (2.23)
Lateral radial vein	4.24 (1.36)	5.18 (2.01)	5.89 (2.43)
Medial radial vein	3.76 (1.25)	4.33 (1.79)	4.82 (2.21)
**Cross-sectional area (mm**^**2**^)			
Radial artery	4.23 (1.75)	3.92 (1.71)	3.90 (1.88)
Lateral radial vein	1.43 (0.83)	1.29 (0.85)	1.25 (0.76)
Medial radial vein	1.58 (0.98)	1.35 (0.84)	1.32 (0.85)

**Table 3:** Primary linear mixed model results for blood vessel depth [primary analysis (N=147)]. P-values in **bold** indicate statistical significance (P < 0.05).

Predictor	Radial Artery		Lateral Radial Vein		Medial Radial Vein	
	$\beta$	P-value	$\beta$	P-value	$\beta$	P-value
Intercept	−0.52	0.272	0.19	0.757	0.01	0.980
Arm Side (Left)	−0.45	<**0.001**	−0.43	<**0.001**	−0.41	<**0.001**
Measurement Position (4 cm)	0.72	<**0.001**	0.99	<**0.001**	0.60	<**0.001**
Measurement Position (6 cm)	1.31	<**0.001**	1.65	<**0.001**	1.09	<**0.001**
Sex (Male)	−1.01	<**0.001**	−1.09	<**0.001**	−1.01	<**0.001**
Age (Years)	0.01	0.278	0.00	0.581	0.01	0.404
BMI (kg/m$^2$)	0.17	<**0.001**	0.18	<**0.001**	0.17	<**0.001**

**Table 4:** Primary linear mixed model results for blood vessel cross-sectional area [primary analysis (N=147)]. P-values in **bold** indicate statistical significance (P < 0.05).

Predictor	Radial Artery		Lateral Radial Vein		Medial Radial Vein	
	$\beta$	P-value	$\beta$	P-value	$\beta$	P-value
Intercept	1.58	**0.004**	0.96	<**0.001**	1.15	<**0.001**
Arm Side (Left)	−0.07	0.239	0.14	**0.012**	−0.03	0.640
Measurement Position (4 cm)	−0.31	<**0.001**	−0.16	**0.011**	−0.23	<**0.001**
Measurement Position (6 cm)	−0.33	<**0.001**	−0.22	<**0.001**	−0.27	<**0.001**
Sex (Male)	1.80	<**0.001**	0.79	<**0.001**	0.88	<**0.001**
Age (Years)	0.02	**0.004**	0.00	0.387	0.00	0.296
BMI (kg/m$^2$)	0.04	**0.048**	−0.00	0.953	−0.00	0.842

**Table 5:** Secondary linear mixed model results (handedness effects) for blood vessel depth [secondary analysis (N=96)]. P-values in **bold** indicate statistical significance (P < 0.05).

Predictor	Radial Artery		Lateral Radial Vein		Medial Radial Vein	
	$\beta$	P-value	$\beta$	P-value	$\beta$	P-value
Intercept	−0.16	0.794	0.59	0.377	0.15	0.812
Arm Side (Left)	−0.38	<**0.001**	−0.39	<**0.001**	−0.35	<**0.001**
Handedness (Left)	0.59	0.096	0.65	0.088	0.58	0.114
Interaction (Dominance)	−0.46	0.080	−0.34	0.231	−0.52	0.061
Measurement Position (4 cm)	0.83	<**0.001**	1.06	<**0.001**	0.68	<**0.001**
Measurement Position (6 cm)	1.42	<**0.001**	1.73	<**0.001**	1.17	<**0.001**
Sex (Male)	−1.09	<**0.001**	−1.10	<**0.001**	−1.00	<**0.001**
Age (Years)	0.01	0.493	0.01	0.591	0.01	0.379
BMI (kg/m$^2$)	0.15	<**0.001**	0.16	<**0.001**	0.15	<**0.001**

**Table 6:** Secondary linear mixed model results (handedness effects) for blood vessel cross-sectional area [secondary analysis (N=96)]. P-values in **bold** indicate statistical significance (P < 0.05).

Predictor	Radial Artery		Lateral Radial Vein		Medial Radial Vein	
	$\beta$	P-value	$\beta$	P-value	$\beta$	P-value
Intercept	1.46	**0.022**	0.89	<**0.001**	1.05	<**0.001**
Arm Side (Left)	−0.06	0.445	0.14	**0.029**	−0.04	0.511
Handedness (Left)	0.02	0.938	−0.06	0.711	−0.31	0.112
Interaction (Dominance)	0.01	0.964	0.04	0.794	0.30	0.068
Measurement Position (4 cm)	−0.31	<**0.001**	−0.17	**0.015**	−0.24	<**0.001**
Measurement Position (6 cm)	−0.32	<**0.001**	−0.22	**0.002**	−0.25	<**0.001**
Sex (Male)	2.04	<**0.001**	0.81	<**0.001**	0.83	<**0.001**
Age (Years)	0.03	**0.004**	0.00	0.603	0.00	0.336
BMI (kg/m$^2$)	0.04	0.075	0.00	0.766	0.00	0.792

**Table 7:** Maximum positron range in water for isotopes used in PET.

	^18^F	^11^C	^13^N	^15^O	^68^Ga	^82^Rb
Maximum range (mm)	2.633	4.456	5.572	9.132	10.273	18.603

**Table 8:** Radial artery depth and cross-sectional area for published ultrasound studies of the wrist.

	This work	Domagała *et al.* (2021)	Lee *et al.* (2016)	Sherrin *et al.* (2023)	Quan *et al.* (2014)
Depth (mm)	3.36 (1.25)	3.88 (0.86)	1.99 (0.99)	2.51 (0.12)	2.5 (1.1)
Cross-sectional area (mm^2^)	4.22 (1.75)	1.60 (0.39)	3.80 (1.38)	4.37 (0.07)	4.15 (1.44)

## Data Availability

The datasets generated during and/or analysed during the current study are available from the corresponding author on reasonable request.
